# Ghost in the Axilla: Luminal-Type Breast Cancer and Occult Sentinel Node Metastasis After Neoadjuvant Chemotherapy

**DOI:** 10.3390/jcm14248658

**Published:** 2025-12-06

**Authors:** Gokay Cetinkaya, Ibrahim Burak Bahcecioglu, Sema Horasan, Osman Bardakci, Mehmet Ali Gulcelik

**Affiliations:** 1 Kartal Dr. Lutfi Kirdar City Hospital, 34862 Istanbul, Turkey; 2 Gulhane Research and Training Hospital, Surgical Oncology Clinic, Health Sciences University, 06010 Ankara, Turkey; dr.ibb@hotmail.com (I.B.B.); semahorasan@gmail.com (S.H.); mehmetali.gulcelik@sbu.edu.tr (M.A.G.); 3 Ali Osman Sonmez Oncology Hospital, 16040 Bursa, Turkey; drosmanbardakci@gmail.com

**Keywords:** breast cancer, sentinel lymph node biopsy, neoadjuvant chemotherapy, axillary ultrasonography, molecular subtype, luminal breast cancer

## Abstract

**Background**: Sentinel lymph node biopsy (SLNB) is the standard axillary staging procedure in clinically node-negative breast cancer but remains invasive, non-therapeutic and increasingly questioned in contemporary de-escalation algorithms. After neoadjuvant chemotherapy (NACT), however, the safety of omitting SLNB solely on the basis of a negative axillary ultrasound (AUS) is uncertain, particularly across molecular subtypes with heterogeneous chemosensitivity. This study evaluated the diagnostic performance of preoperative AUS after NACT and explored clinicopathological and biological factors associated with SLNB positivity in ultrasound-negative axillae. **Methods**: In this single-centre retrospective cohort, 135 women with invasive breast cancer who received NACT followed by surgery (2022–2024) were analysed. To avoid spectrum bias, 77 patients with clipped, cytologically or histologically proven node-positive disease at baseline were excluded from the main analysis. All patients underwent preoperative AUS and definitive axillary staging. Ninety-six women with ultrasound-negative axillae who proceeded to SLNB constituted the primary study population. Oestrogen receptor (ER), progesterone receptor (PR), HER2, Ki-67 and immunohistochemistry-based molecular subtype were recorded. Receiver operating characteristic (ROC) analysis and uni/multivariable logistic regression were used as exploratory tools to identify factors associated with SLNB positivity. **Results**: In the overall cohort, AUS sensitivity, specificity, negative predictive value and false-negative rate for axillary metastasis were 47.8%, 90.9%, 62.5% and 52.2%, respectively. Among ultrasound-negative axillae, SLNB was positive in 37.5%. Compared with SLNB-negative patients, those with SLNB metastases more frequently harboured an intratumoural ductal carcinoma in situ (DCIS) component, showed higher ER/PR expression and lower Ki-67, and were predominantly luminal A or luminal B/HER2−, whereas AUS performance appeared more favourable in HER2-enriched and triple-negative tumours. ROC-derived cut-offs for ER (82.5%), PR (25.0%) and Ki-67 (17.5%) provided only moderate discrimination (area under the curve 0.68–0.70). In multivariable analysis, absence of a DCIS component and low PR expression were independently associated with reduced odds of SLNB positivity, suggesting that DCIS and high PR may act as indicators of residual nodal risk in ultrasound-negative axillae. All estimates are limited by sample size and wide confidence intervals and should be interpreted as hypothesis-generating. **Conclusions**: Preoperative AUS alone cannot reliably exclude sentinel lymph node metastasis after NACT, particularly in luminal A and luminal B/HER2− tumours with strong hormone receptor expression and a low proliferative index. Until prospective, biology-stratified trials confirm the safety of omission, SLNB should not be withheld solely on the basis of a negative AUS in these subtypes. Axillary management after NACT should systematically integrate both imaging findings and tumour biology when considering further de-escalation of surgery.

## 1. Introduction

Breast cancer is the most frequently diagnosed malignancy and a leading cause of cancer-related mortality among women worldwide, with more than two million new cases and over 680,000 deaths estimated in 2020. These figures from GLOBOCAN 2020 highlight both the global burden and the ongoing need to optimise staging and treatment strategies in this biologically heterogeneous disease [1]. The increasing use of population-based screening and advances in imaging have shifted the stage distribution towards smaller, earlier-stage tumours, so that a substantial proportion of women now present with clinically node-negative (cN0) disease at diagnosis [1,2]. In this setting, axillary nodal status remains one of the strongest prognostic determinants and continues to influence decisions regarding systemic therapy and regional radiotherapy [3,4,5]. Parallel efforts to refine prognostication using multigene and molecular models further emphasise the central role of accurate staging in individualised care [6].

Historically, axillary lymph node dissection (ALND) was routinely performed in invasive breast cancer, providing definitive pathological staging but at the cost of considerable long-term morbidity, including lymphoedema, sensory neuropathy and shoulder dysfunction [4,5,7,8]. The introduction of sentinel lymph node biopsy (SLNB) represented a major step in surgical de-escalation of the axilla. Large prospective trials and subsequent reviews established SLNB as the standard staging procedure in clinically and radiologically node-negative patients, with substantially lower morbidity than ALND and excellent staging accuracy [8,9,10,11,12,13]. Nevertheless, SLNB remains an invasive operation that may cause seroma, wound infection, allergic reactions to tracers and chronic arm symptoms, and it confers no direct therapeutic benefit beyond staging [7,9,10,11]. Together with evidence from ACOSOG Z0011 and AMAROS demonstrating that completion ALND can safely be omitted in selected patients with limited nodal involvement who receive appropriate systemic therapy and radiotherapy [8,13], these considerations have prompted the further question of whether SLNB itself can be safely omitted in carefully selected low-risk patients [14,15,16].

High-resolution axillary ultrasonography (AUS) is widely used as a non-invasive, readily available and cost-effective tool for preoperative assessment of nodal status. AUS evaluates morphologic features such as cortical thickness, eccentric or diffuse cortical hypertrophy, loss of the fatty hilum and altered nodal shape and, when combined with ultrasound-guided fine-needle aspiration or core biopsy (US-FNA/CB), can reliably identify patients with a high axillary tumour burden who are unlikely to be candidates for axillary de-escalation [12,17,18,19,20]. Across multiple retrospective series and meta-analyses, AUS and AUS-guided needle sampling have consistently demonstrated high specificity and positive predictive value for macrometastatic nodal disease, but only modest sensitivity and negative predictive value, particularly for micrometastases and small-volume involvement [3,12,17,18,19,20]. In early-stage cohorts undergoing primary surgery, false-negative AUS findings are therefore not uncommon, implying that a relevant proportion of node-positive patients will have apparently “normal” or “reactive” axillary ultrasound and will only be detected by SLNB [3,17,18,19,20].

Against this background, several contemporary de-escalation trials have directly examined whether a radiologically negative axilla on AUS is sufficient to omit SLNB in selected patients undergoing upfront surgery. The SOUND (Sentinel node vs. Observation after Axillary UltraSouND) randomised clinical trial compared SLNB with no axillary surgery in women with tumours ≤ 2 cm, cN0 axilla and negative AUS, and demonstrated non-inferiority of omitting SLNB with respect to 5-year distant disease-free survival, with very low axillary failure rates in both arms [21]. Parallel studies such as INSEMA, BOOG 2013-08 and NAUTILUS are similarly evaluating the oncologic safety of omitting SLNB in cN0 patients with negative axillary imaging who undergo breast-conserving surgery [22,23,24]. Emerging data from these trials, together with expert commentaries and clinical reviews, support the concept that SLNB may be unnecessary in selected patients with favourable tumour characteristics and negative AUS, and editorials have begun to question whether SLNB is still required in all patients with a radiologically negative axilla [14,15,16,21,22,23,24].

However, the evidence from these de-escalation trials largely pertains to patients undergoing upfront surgery and cannot be directly extrapolated to those treated with neoadjuvant chemotherapy (NACT). In the NACT setting, post-treatment axillary status reflects both the initial tumour burden and the degree of treatment response. Therapy-induced fibrosis, architectural distortion and treatment-related changes in nodal size and morphology may all alter the sonographic appearance and detectability of residual disease, and these effects may vary across biological subtypes. Studies in initially node-positive cohorts, including ACOSOG Z1071, indicate that imaging-based selection strategies after NACT—such as post-treatment AUS and targeted axillary approaches—can reduce the false-negative rate of SLNB by enriching for patients with nodal pathological complete response (pCR), but residual microscopic disease remains a persistent problem [25,26]. Robust data on the negative predictive value of AUS specifically in clinically or sonographically node-negative axillae after NACT, and on how this performance varies by tumour biology, remain limited.

Breast cancer is biologically heterogeneous, and intrinsic molecular subtypes derived from gene-expression profiling—luminal A, luminal B, HER2-enriched and basal-like/triple-negative—have distinct patterns of recurrence and responsiveness to systemic therapy [27,28]. Immunohistochemistry-based surrogates using oestrogen receptor (ER), progesterone receptor (PR), HER2 and the Ki-67 proliferation index are widely used in routine practice to approximate these subtypes [27,28]. Luminal A and luminal B/HER2-negative tumours, characterised by strong hormone receptor expression and relatively low proliferation, generally exhibit more favourable long-term survival but lower breast and nodal pCR rates after NACT than HER2-enriched and triple-negative subtypes, which are more chemosensitive yet biologically aggressive [29,30,31]. Large pooled analyses and subtype-specific neoadjuvant trials have shown that pCR is both more frequent and more strongly associated with improved event-free and overall survival in triple-negative and HER2-positive disease than in hormone receptor-positive, HER2-negative tumours [29,30,31]. These biological differences are likely to influence both the pattern of residual axillary disease and its sonographic visibility after NACT, raising the possibility of particularly high false-negative AUS rates in luminal tumours that fail to achieve nodal pCR [25,26,29,30,31].

In this context, combining preoperative AUS with readily available pathological markers could refine risk stratification for occult axillary metastasis in patients receiving NACT. Identifying subgroups in whom SLNB could be safely omitted, versus those in whom omission would carry an unacceptably high risk of missed nodal disease, would have immediate implications for surgical planning and regional radiotherapy decisions [14,15,16,21]. Yet, no widely accepted biology-integrated algorithm currently exists to guide omission of SLNB in the post-NACT setting.

The aim of the present study was therefore to evaluate the diagnostic performance of preoperative AUS for detecting axillary metastasis after NACT in a cohort of 135 women with breast cancer and to explore clinicopathological and biological predictors of SLNB positivity among the 96 patients with ultrasound-negative axillae. We focused on hormone receptor status, HER2 expression, Ki-67 index and immunohistochemistry-based molecular subtypes, using ROC-derived thresholds and uni and multivariable logistic regression models. We hypothesised that luminal tumours with high ER/PR expression and low Ki-67 would be associated with higher rates of false-negative AUS and occult sentinel node metastases, thereby defining a subgroup in which SLNB should not be omitted despite negative axillary imaging.

## 2. Materials and Methods

### 2.1. Patient Population

This retrospective, single-centre cohort study included women with histologically confirmed invasive breast carcinoma who received NACT followed by breast-conserving surgery or mastectomy at the Surgical Oncology Clinic of the University of Health Sciences, Ankara Gulhane Training and Research Hospital, between January 2022 and December 2024. Medical records of 212 consecutive patients were screened.

Because the primary objective was to evaluate the diagnostic performance of preoperative AUS and to identify predictors of SLNB positivity in patients with a sonographically negative axilla, predefined exclusion criteria were applied. Seventy-seven women had biopsy-proven metastatic axillary lymph nodes at baseline that were marked with a clip before NACT. These patients were excluded from the present analysis to avoid spectrum bias and overestimation of AUS diagnostic performance, as their inclusion would have enriched the cohort with node-positive disease not representative of the clinically/sonographically node-negative population of interest.

Patients were also excluded if any of the following were missing or incomplete: preoperative AUS report, final nodal pathology following SLNB and/or ALND or immunohistochemical data on ER, PR, HER2 or Ki-67. After application of all criteria, 135 women with complete clinicopathological, imaging and pathological information comprised the study cohort used to assess the diagnostic performance of preoperative AUS.

Within this cohort, 96 patients had a preoperative AUS classified as reactive/non-suspicious (ultrasound-negative axilla), whereas 39 patients had at least one lymph node categorised as pathologic/suspicious. The subgroup of 96 women with ultrasound-negative axillae who underwent SLNB formed the analytic population for ROC analyses and logistic regression models of predictors of SLNB positivity.

### 2.2. Study Design, Imaging Assessment and Data Collection

The study protocol was approved by the Ethics Committee of the University of Health Sciences, Ankara Gulhane Training and Research Hospital, and the study was conducted in accordance with the Declaration of Helsinki. Owing to the retrospective design and anonymised data extraction, the requirement for written informed consent was waived by the ethics committee.

Clinical, radiological and pathological data were retrieved from electronic medical records, radiology reports and operative/pathology charts using a standardised abstraction form. For each eligible patient, the following variables were recorded: age at diagnosis; tumour laterality and localisation; histological type and grade; presence of an associated ductal carcinoma in situ (DCIS) component; presence of lymphovascular invasion (LVI); tumour size on imaging and on final pathology; Nottingham histological score; mammographic findings; axillary ultrasound findings; type of breast and axillary surgery; and details of adjuvant systemic therapies and radiotherapy.

Final axillary nodal status was obtained from SLNB and/or ALND pathology reports and included the total number of examined sentinel and non-sentinel lymph nodes and the number of nodes with metastasis. In all analyses, SLNB positivity was defined as the presence of at least one metastatic sentinel node on final histopathology, including macrometastases and micrometastases; isolated tumour cells, when present, were not considered as SLNB-positive for the primary analyses.

Preoperative AUS was performed as part of routine staging by experienced breast radiologists using high-frequency linear-array transducers. All accessible axillary levels were systematically evaluated. Lymph nodes were categorised as reactive/non-suspicious or pathologic/suspicious according to prespecified morphologic criteria. Reactive/non-suspicious nodes were characterised by an oval or reniform shape, preserved fatty hilum, thin and homogeneous cortex and a normal hilar vascular pattern. Pathologic/suspicious nodes were defined by one or more of the following features: cortical thickening (focal or diffuse), eccentric cortical hypertrophy, loss or displacement of the fatty hilum, round configuration and/or abnormal non-hilar or peripheral vascularisation on colour Doppler imaging.

In patients with AUS-suspicious nodes, ultrasound-guided fine-needle aspiration or core needle biopsy was performed according to institutional practice, and cytology/histology results were recorded when available. After completion of NACT, axillary surgery was planned within a multidisciplinary tumour board. Patients with an ultrasound-negative axilla routinely underwent SLNB. Patients with persistent AUS-suspicious or biopsy-proven nodal disease after NACT were treated with SLNB and/or ALND at the discretion of the treating team, taking into account baseline nodal status, treatment response and contemporary guideline recommendations.

For the overall cohort of 135 patients, the relationship between preoperative AUS category (reactive/non-suspicious vs. pathologic/suspicious) and final pathological nodal status was used to derive sensitivity, specificity, positive predictive value (PPV), negative predictive value (NPV), false-negative rate (FNR) and overall diagnostic accuracy of AUS.

Within the subgroup of 96 women with ultrasound-negative axillae, more detailed analyses were undertaken to explore factors associated with SLNB positivity. In this analytic cohort, we examined associations between SLNB status and clinicopathological variables (age, tumour size, DCIS component, LVI and Nottingham histological score), biomarker expression (ER, PR, HER2 and Ki-67) and intrinsic molecular subtype. ROC-curve analyses of ER, PR and Ki-67 were performed to evaluate their ability to discriminate SLNB-positive from SLNB-negative patients, and univariable and multivariable binary logistic regression models were constructed to estimate odds ratios (ORs) and 95% confidence intervals (CIs) for SLNB positivity.

### 2.3. Immunohistochemistry and Classification of Molecular Subtypes

All histopathological evaluations were performed in the institutional pathology department according to standard protocols. Immunohistochemistry (IHC) for ER, PR, HER2 and Ki-67 was assessed on core biopsy or surgical specimens as part of routine diagnostic work-up.

For ER, PR and Ki-67, nuclear staining of invasive tumour cells was evaluated, and the percentage of positively stained nuclei was reported. HER2 status was assessed by membranous staining intensity and completeness, using a semi-quantitative scoring system in line with institutional and contemporary guideline practice; equivocal (2+) cases were further evaluated by in situ hybridisation where clinically indicated, and HER2 status was ultimately dichotomised as positive or negative for analysis. The Ki-67 proliferation index was expressed as the percentage of positive nuclei among at least 500 invasive tumour cells in hot-spot areas, and a threshold of 14% was used to classify Ki-67 as low (<14%) or high (≥14%), consistent with St. Gallen-based recommendations.

Based on IHC results, tumours were assigned to intrinsic surrogate molecular subtypes as follows:

- Luminal A: ER-positive and/or PR-positive, HER2-negative and low Ki-67 (<14%).
- Luminal B/HER2-negative: ER-positive and/or PR-positive, HER2-negative and high Ki-67 (≥14%).
- Luminal B/HER2-positive: ER-positive and/or PR-positive and HER2-positive, irrespective of Ki-67.
- HER2-enriched: ER-negative, PR-negative and HER2-positive.
- Basal-like (triple-negative): ER-negative, PR-negative and HER2-negative.

These molecular subtype categories were used to compare the diagnostic performance of AUS across biologically distinct groups and were also included as covariates in logistic regression models assessing predictors of SLNB positivity among patients with ultrasound-negative axillae.

### 2.4. Statistical Analysis

All statistical analyses were performed using IBM SPSS Statistics for Windows, version 27.0 (IBM Corp., Armonk, NY, USA). Continuous variables were examined for normality using the Kolmogorov–Smirnov and Shapiro–Wilk tests and for homogeneity of variance using Levene’s test. Normally distributed variables are presented as mean ± standard deviation (SD) and were compared between SLNB-negative and SLNB-positive groups using the independent-samples Student’s *t*-test. Non-normally distributed variables are presented as median (interquartile range, IQR) and were compared using the Mann–Whitney U test. Categorical variables are expressed as counts and percentages and were compared using Pearson’s χ^2^ test or Fisher’s exact test, as appropriate.

The diagnostic performance of preoperative AUS was evaluated using 2 × 2 contingency tables, with final histopathologic nodal status (node-negative vs. node-positive) as the reference standard. From these tables, sensitivity, specificity, PPV, NPV and FNR were calculated using conventional definitions.

In the ultrasound-negative subgroup (*n* = 96), ROC-curve analysis was used to assess the discriminatory ability of ER, PR and Ki-67 for SLNB positivity. The area under the ROC curve (AUC) and corresponding 95% CIs were estimated using non-parametric methods. For each biomarker, the optimal cut-off value was determined by maximising the Youden index (sensitivity + specificity − 1), and these thresholds were subsequently used to dichotomise ER, PR and Ki-67 into “low” and “high” categories in regression analyses.

Associations between clinicopathological/biological variables and SLNB positivity were first explored using univariable binary logistic regression, with results reported as ORs and 95% CIs. Variables with a *p* value < 0.10 in univariable analyses were entered simultaneously into a multivariable binary logistic regression model (enter method) to identify independent predictors of SLNB positivity. Model calibration was evaluated using the Hosmer–Lemeshow goodness-of-fit test, and explanatory power was summarised using the Cox & Snell and Nagelkerke R^2^ statistics; overall classification accuracy was also recorded. All statistical tests were two-sided, and a *p* value < 0.05 was considered statistically significant.

## 3. Results

### 3.1. Patient Characteristics

A total of 135 female patients met the eligibility criteria and were included in the analysis. The mean age at diagnosis was 48.49 ± 11.12 years (range, 27–82). At diagnosis, 75 patients (55.6%) were postmenopausal, whereas 60 (44.4%) were premenopausal and subsequently developed chemotherapy-induced amenorrhoea during or after NACT. Tumours arose in the left breast in 67 patients (49.6%), in the right breast in 63 (46.7%) and bilaterally in 5 (3.7%). Lesions were unifocal in 114 patients (84.4%) and multifocal in 21 (15.6%).

Invasive ductal carcinoma (IDC) with an associated ductal carcinoma in situ (DCIS) component was the most frequent histopathological pattern (*n* = 62, 45.9%), followed by pure IDC (*n* = 55, 40.7%). Invasive lobular carcinoma (ILC) and ILC with DCIS were less common (*n* = 3, 2.2% and *n* = 4, 3.0%, respectively), while other histologies accounted for 11 cases (8.1%). Overall, a DCIS component was present in 61 patients (45.2%). Microcalcification clusters on imaging were documented in 76 patients (56.3%).

Breast-conserving surgery (BCS) was performed in 70 patients (51.9%), whereas 65 (48.1%) underwent mastectomy. For definitive axillary management, 75 patients (55.6%) were treated with SLNB alone and 60 (44.4%) underwent ALND, with or without preceding SLNB. Intraoperative frozen-section examination of SLNs was negative in 77 patients (57.0%) and paraffin-section analysis was negative in 66 (48.9%). The mean number of SLNs examined was 3.87 ± 1.73 on frozen section and 4.77 ± 2.39 on paraffin section. The mean number of metastatic SLNs (including micro and macrometastases) was 0.70 ± 1.03 on frozen section and 0.90 ± 1.26 on paraffin section. Among patients who underwent ALND, a mean of 16.10 ± 7.08 lymph nodes were removed, of which 3.60 ± 3.78 were metastatic on average.

With respect to biomarker expression, ER positivity was observed in 98 patients (72.5%) and PR positivity in 91 (67.4%). The mean ER and PR expression levels were 59.61 ± 39.62% and 28.84 ± 33.12%, respectively, and the mean Ki-67 proliferation index was 28.07 ± 25.53%. HER2 was negative in 108 patients (80.0%) and positive in 27 (20.0%). According to immunohistochemistry-defined intrinsic subtypes, 53 patients (39.3%) were classified as luminal A, 34 (25.2%) as luminal B/HER2−, 11 (8.1%) as luminal B/HER2+, 11 (8.1%) as HER2-enriched and 26 (19.3%) as triple-negative. No patient had distant organ metastasis at the time of diagnosis.

Radiologic–pathologic concordance of the axilla, defined as agreement between preoperative AUS category (reactive/non-suspicious vs. pathologic/suspicious) and final histopathologic nodal status (negative vs. positive), was present in 93 patients (68.9%) and absent in 42 (31.1%). Baseline demographic and clinicopathologic characteristics of the cohort are summarised in Table 1.

### 3.2. Clinicopathological Comparison According to SLNB Status in Patients with Ultrasound-Negative Axilla

Among the 135 patients, 96 (71.1%) had a preoperative AUS classified as reactive/non-suspicious and underwent SLNB. Within this ultrasound-negative subgroup, 60 patients (62.5%) had negative SLNB (SLNB− group) and 36 (37.5%) had at least one metastatic SLN (SLNB+ group). Clinicopathological and biological characteristics of these two groups are summarised in Table 2.

There was no significant difference in age between SLNB− and SLNB+ patients (median 45 vs. 45 years, *p* = 0.124). Tumour laterality, lesion pattern (solitary vs. multiple), histopathological type, tumour size on ultrasound and final pathology, presence of lymphovascular invasion and the number of SLNs removed did not differ significantly between the groups (all *p* > 0.05).

In contrast, the presence of a DCIS component was significantly more frequent in SLNB+ than in SLNB− patients (63.9% vs. 26.7%, *p* < 0.001). Microcalcification clusters were also more common in the SLNB+ group, although this difference did not reach conventional statistical significance (69.4% vs. 50.0%, *p* = 0.062).

The median Nottingham histological score was lower in the SLNB+ group than in the SLNB− group (6 vs. 7, *p* = 0.005), indicating that axillary metastases occurred not only in morphologically high-grade tumours. Regarding biomarker expression, ER and PR levels were significantly higher in SLNB+ patients (median ER 90% vs. 70%, *p* = 0.003; median PR 40% vs. 1.5%, *p* = 0.003), whereas the Ki-67 index was significantly lower (median 10% vs. 35%, *p* = 0.001). HER2 negativity was more frequent in the SLNB+ group (91.7% vs. 66.7%, *p* = 0.005).

When patients were stratified by molecular subtype, luminal A and luminal B/HER2− tumours were over-represented in the SLNB+ group (58.3% and 27.8%, respectively) compared with the SLNB− group (28.3% and 16.7%; overall *p* = 0.003). Conversely, triple-negative and HER2-enriched tumours were more frequent in the SLNB− group, consistent with higher chemosensitivity and lower residual nodal involvement in these subtypes.

### 3.3. Diagnostic Performance of Preoperative Axillary Ultrasound in the Entire Cohort and According to Molecular Subtype

The diagnostic performance of preoperative AUS for detecting axillary metastasis in the entire cohort is shown in Table 3A. Of 135 patients, 96 (71.1%) had axillae categorised as reactive/non-suspicious and 39 (28.9%) as pathologic/suspicious. On final pathology, 69 patients (51.1%) had nodal metastasis. AUS correctly identified 33 of these node-positive cases, yielding a sensitivity of 47.8% (33/69). Of 66 node-negative patients, 60 were correctly classified as reactive, resulting in a specificity of 90.9% (60/66).

The PPV of a pathologic/suspicious AUS finding was 84.6% (33/39), whereas the NPV of a reactive/non-suspicious AUS was 62.5% (60/96). Consequently, the FNR of AUS—that is, the proportion of node-positive patients with a reactive/non-suspicious AUS—was 52.2% (36/69). The association between AUS findings and final nodal status was statistically significant (Pearson’s χ^2^ = 24.64, df = 1, *p* < 0.001).

When analysed by molecular subtype (Table 3B), sensitivity was lowest in luminal A tumours (40.0%) and luminal B/HER2− tumours (54.5%), with corresponding FNRs of 60.0% and 45.5%, respectively. In these luminal subtypes, NPV was also limited (44.7% in luminal A and 50.0% in luminal B/HER2−), despite high specificity (94.4% and 83.3%, respectively). By contrast, AUS performance was more favourable in HER2-enriched and triple-negative tumours, with sensitivities of 66.7% and 57.1% and NPVs of 88.9% and 84.2%, respectively, although FNRs remained non-negligible (33.3% and 42.9%).

Overall, preoperative AUS showed high specificity and PPV across molecular subtypes but particularly poor sensitivity and high FNRs in luminal A and luminal B/HER2− tumours, which constituted the main contributors to occult nodal disease in the ultrasound-negative axilla.

### 3.4. ROC-Curve Analysis of ER, PR and Ki-67 for Predicting SLNB Positivity in Ultrasound-Negative Axilla

**Figure 1:**
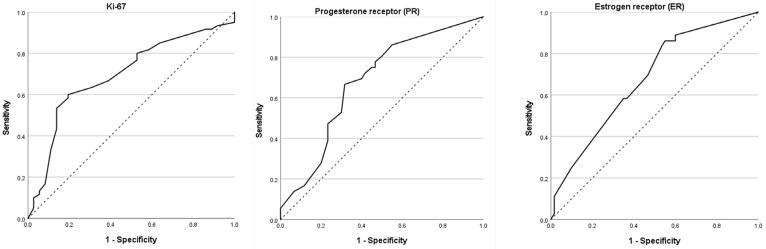
ROC-Curve analysis of ER, PR and Ki-67 for SLNB metastases in ultrasonographic negative patients. Receiver operating characteristic (ROC) curves show the ability of each biomarker to predict sentinel lymph node biopsy (SLNB) metastasis in patients with ultrasound-negative axillae (*N* = 96). The diagonal dotted line represents the reference line (AUC = 0.5). Abbreviations: ROC, receiver operating characteristic; AUC, area under the curve; ER, oestrogen receptor; PR, progesterone receptor; Ki-67, proliferation index; SLNB, sentinel lymph node biopsy.

In the ultrasound-negative subgroup (*n* = 96), ROC-curve analysis was used to evaluate the ability of ER, PR and Ki-67 to predict SLNB positivity (Figure 1, Table 4). All three biomarkers demonstrated statistically significant, but only moderate, discriminative performance.

For ER, the AUC was 0.679 (95% CI, 0.571–0.787; *p* = 0.003). An optimal cut-off of 82.5% ER-positive tumour cells, derived from the Youden index, yielded a sensitivity of 58.3% and a specificity of 63.3%. PR demonstrated an identical AUC of 0.679 (95% CI, 0.571–0.788; *p* = 0.003). At the optimal cut-off of 25.0% PR-positive cells, sensitivity was 66.7% and specificity 68.3%. Ki-67 achieved the highest AUC among the three markers (0.696; 95% CI, 0.587–0.805; *p* = 0.001). A cut-off of 17.5% for Ki-67 yielded a sensitivity of 63.3% and a specificity of 69.4%.

These data indicate that higher ER and PR expression and lower Ki-67 index are associated with an increased likelihood of SLNB positivity in patients with an ultrasound-negative axilla. However, given the moderate AUC values and the relatively small sample size, none of these markers alone provides high diagnostic accuracy, supporting their use as adjunctive rather than stand-alone tools in risk stratification.

### 3.5. Predictors of SLNB Positivity in Patients with Ultrasound-Negative Axilla

Predictors of SLNB positivity in the ultrasound-negative subgroup (*n* = 96) were first evaluated using univariable logistic regression (Table 5A). Age and pathological tumour size, treated as continuous variables, were not significantly associated with SLNB status (per 1-year increase: OR 1.04, 95% CI 0.99–1.08; *p* = 0.097; per 1 mm increase: OR 1.01, 95% CI 0.98–1.04; *p* = 0.743).

The absence of a DCIS component was associated with substantially lower odds of SLNB positivity compared with the presence of DCIS (OR 0.21, 95% CI 0.09–0.50; *p* < 0.001). Microcalcification clusters tended to be less frequent in SLNB− patients, with an OR of 0.44 (95% CI 0.18–1.05; *p* = 0.065). Lymphovascular invasion was not significantly associated with SLNB outcome (OR 1.17, 95% CI 0.51–2.68; *p* = 0.711). Interestingly, a lower Nottingham histological score was associated with higher odds of SLNB positivity (OR 3.27, 95% CI 1.38–7.74; *p* = 0.007), suggesting that nodal metastases were not restricted to morphologically high-grade tumours.

When analysed using ROC-derived thresholds, “low” ER expression (<82.5%) was associated with reduced odds of SLNB positivity compared with “high” ER (≥82.5%) (OR 0.41, 95% CI 0.18–0.96; *p* = 0.041). This association was even more pronounced for PR: low PR (<25.0%) was associated with markedly lower odds of SLNB metastasis than high PR (≥25.0%) (OR 0.23, 95% CI 0.10–0.56; *p* = 0.001). Consistent with the descriptive analyses, tumours with a low Ki-67 index (≤17.5%) were more likely to be SLNB-positive than those with high Ki-67 (>17.5%), with an OR of 3.93 (95% CI 1.62–9.49; *p* = 0.002). In keeping with the higher prevalence of HER2 negativity in the SLNB+ group, HER2-negative tumours had approximately 5.5-fold higher odds of SLNB positivity compared with HER2-positive tumours (OR 5.50, 95% CI 1.50–20.14; *p* = 0.010).

In a separate univariable model using molecular subtype (reference category: triple-negative), both luminal A and luminal B/HER2− tumours were associated with significantly higher odds of SLNB positivity (OR 6.59, 95% CI 1.64–26.43; *p* = 0.008 and OR 5.33, 95% CI 1.18–24.21; *p* = 0.030, respectively), whereas luminal B/HER2+ and HER2-enriched subtypes did not differ significantly from triple-negative disease (both *p* > 0.60).

Variables with *p* < 0.10 in univariable analyses (DCIS component, microcalcification cluster, ER, PR, Ki-67) were entered into a multivariable logistic regression model (Table 5B). The final model demonstrated good calibration (Hosmer–Lemeshow χ^2^ = 0.304, df = 7, *p* = 1.000) and explained approximately 30% of the variance in SLNB status (Nagelkerke R^2^ = 0.304), with an overall correct classification rate of 71.9%.

After adjustment, absence of a DCIS component remained independently associated with lower odds of SLNB positivity (adjusted OR 0.33, 95% CI 0.12–0.91; *p* = 0.033). Low PR expression (<25.0%) also emerged as an independent protective factor (adjusted OR 0.30, 95% CI 0.11–0.81; *p* = 0.017). Low Ki-67 (≤17.5%) retained a positive association with SLNB metastasis (adjusted OR 2.60, 95% CI 0.96–7.05; *p* = 0.060), although this did not reach conventional statistical significance, reflecting limited sample size and wide confidence intervals. Microcalcification clusters and ER expression were not independently associated with SLNB status (both *p* > 0.60).

Taken together, these findings indicate that, among patients with ultrasound-negative axillae, the presence of a DCIS component and high PR expression are independent indicators of increased risk of sentinel lymph node metastasis, consistent with the higher false-negative AUS rates observed in luminal tumours. Ki-67 may provide additional prognostic information but should be interpreted cautiously given the modest discriminative performance and the small sample size.

## 4. Discussion

In this retrospective single-centre cohort of breast cancer patients treated with NACT, we evaluated the performance of preoperative AUS and examined how tumour biology modifies the risk of sentinel lymph node metastasis in ultrasound-negative axillae. In the overall cohort of 135 patients, AUS demonstrated high specificity (90.9%) but only moderate sensitivity (47.8%) for detecting axillary metastasis, resulting in a false-negative rate of 52.2% and an NPV of 62.5%. Among the 96 patients with ultrasound-negative axillae who proceeded to SLNB, more than one-third (37.5%) harboured nodal metastases. Within this ultrasound-negative subgroup, SLNB positivity was strongly associated with the presence of an intratumoural DCIS component, higher ER and PR expression, lower Ki-67 index and luminal A or luminal B/HER2-negative molecular subtype. ROC-derived cut-off values for ER (82.5%), PR (25.0%) and Ki-67 (17.5%) yielded AUCs in the 0.68–0.70 range, indicating only moderate discrimination. In multivariable analysis, absence of DCIS and low PR expression remained independently associated with lower odds of SLNB positivity, whereas low Ki-67 showed a borderline significant trend towards higher odds of nodal metastasis. Taken together, these findings indicate that ultrasound-negative axillae after NACT constitute a biologically heterogeneous group in which luminal tumours with strong hormone receptor expression and low proliferation carry a substantial risk of occult nodal disease despite negative imaging.

### 4.1. Diagnostic Performance of Axillary Ultrasound After Neoadjuvant Chemotherapy

Our estimates of AUS performance in the entire cohort are broadly consistent with reports from early-stage breast cancer populations undergoing primary surgery, although the false-negative rate in our NACT-treated cohort is even higher. In non-NACT cohorts, AUS sensitivities typically range from 50 to 70% and specificities from 80 to 95% for nodal metastasis, with performance improving when AUS is combined with US-guided needle sampling [3,12,17,18,19,20]. Rukanskienė et al. reported sensitivity and specificity values of 37.5% and 95.1%, respectively, for AUS compared with SLNB in early breast cancer, with an NPV of 77.3%, illustrating the high specificity but modest sensitivity of AUS [3]. Similar results have been observed in large audits and meta-analyses, in which AUS and AUS-guided biopsy reliably identify patients with extensive nodal disease but miss a significant proportion of those with low-volume involvement [18,19,20].

In the NACT setting, the diagnostic landscape is more complex. In initially node-positive patients, neoadjuvant studies have shown that AUS performed after NACT can reduce the false-negative rate of SLNB by enriching for patients with nodal pCR, but residual disease persists in a subset of patients with negative or equivocal AUS findings [25,26]. Other neoadjuvant studies and pooled analyses similarly suggest that AUS- and US-guided biopsy can help tailor axillary surgery after NACT, particularly in cN+ cohorts, but they provide limited information on AUS as a rule-out test in patients who are clinically and sonographically node-negative after systemic therapy [25,26,29].

In our cohort, AUS correctly classified most histologically node-negative patients yet missed more than half of those with residual nodal metastasis, resulting in a false-negative rate of 52.2% and an NPV of 62.5%. From a de-escalation perspective, these values are unlikely to be acceptable as the sole basis for omitting SLNB in NACT-treated patients. Moreover, our subtype-stratified analyses underscore that this limitation is not uniform across biological groups. Sensitivity and NPV were particularly poor in luminal A and luminal B/HER2-negative tumours, with false-negative rates exceeding 45–60%, whereas performance was more favourable in HER2-enriched and triple-negative subtypes.

These patterns are biologically plausible. HER2-positive and triple-negative tumours generally achieve higher breast and nodal pCR rates under contemporary systemic regimens and may leave less small-volume residual nodal disease, whereas luminal tumours often retain microscopic metastases that are sonographically subtle and difficult to distinguish from reactive nodes [27,28,29,30,31]. From a clinical standpoint, our data suggest that in the post-NACT setting a negative AUS cannot currently be considered sufficient to omit SLNB, particularly in luminal subtypes. At the same time, it should be noted that our findings address diagnostic performance and do not, by themselves, establish that all false-negative cases would translate into clinically meaningful differences in recurrence or survival in the context of modern systemic therapy and regional irradiation.

### 4.2. Molecular Subtype, Hormone Receptor Expression and Ki-67 as Predictors of Occult Nodal Disease

A major strength of our study is the integration of detailed immunohistochemical data with axillary imaging in the AUS-negative subgroup. Using ROC-curve analysis, we identified cut-off values of 82.5% for ER, 25.0% for PR and 17.5% for Ki-67 that provided moderate ability to discriminate between SLNB-positive and SLNB-negative patients. While none of these markers alone achieved high diagnostic accuracy, their associations with SLNB outcome were clinically coherent and consistent with known biology.

In univariable logistic regression, low ER and PR expression—defined by values below the ROC-derived thresholds—were associated with substantially lower odds of SLNB positivity compared with high expression, whereas tumours with a low Ki-67 index (≤17.5%) paradoxically exhibited higher odds of nodal metastasis than those with high Ki-67. These findings align with the established patterns of response across intrinsic subtypes: luminal A and luminal B/HER2-negative tumours, characterised by strong hormone receptor expression and relatively low proliferation, frequently show incomplete nodal response to NACT and may harbour residual micrometastatic disease despite modest radiologic changes [27,28,29,30,31]. In contrast, HER2-enriched and triple-negative tumours demonstrate higher pCR rates, including nodal pCR, when effective systemic therapy—such as dual HER2 blockade or intensive anthracycline/taxane-based regimens—is administered, thereby reducing the pool of residual nodal disease [29,30,31].

Importantly, the presence of an intratumoural DCIS component emerged as a strong imaging-independent predictor of SLNB positivity. DCIS was significantly more frequent in the SLNB-positive group than in the SLNB-negative group, and the absence of DCIS was associated with an approximately three- to four-fold reduction in the odds of nodal metastasis in univariable analysis. In the multivariable model adjusting for ER, PR, Ki-67 and microcalcification clusters, absence of DCIS and low PR expression remained independently associated with lower odds of SLNB positivity, whereas the association with low Ki-67 retained a positive direction but fell short of conventional statistical significance. The mechanistic explanation for the relationship between DCIS and nodal involvement remains speculative but may reflect a larger overall disease burden, a more extensive intraductal component or biological features that favour persistence of low-volume nodal disease despite NACT.

Taken together, these results suggest that the combination of luminal subtype, high ER/PR expression, low Ki-67 index and intratumoural DCIS defines a phenotype with a high risk of occult sentinel node metastases despite negative AUS. Conversely, triple-negative and HER2-enriched tumours in our cohort showed lower SLNB positivity rates and more favourable AUS performance, consistent with their higher chemosensitivity and nodal pCR rates. These patterns do not imply that SLNB can yet be omitted in these subtypes, but they help to delineate the biological spectrum of risk within ultrasound-negative axillae and identify subgroups in which future de-escalation strategies may be prioritised.

### 4.3. Context Within Ongoing De-Escalation of Axillary Surgery

Trials such as ACOSOG Z0011 and AMAROS have already transformed axillary management by demonstrating that ALND can be safely omitted in selected patients with limited sentinel node involvement who receive appropriate systemic therapy and radiotherapy [8,13]. Building on this, SOUND, INSEMA, BOOG 2013-08 and NAUTILUS are now testing whether SLNB itself can be omitted in clinically node-negative, imaging-negative patients undergoing upfront breast-conserving surgery [14,15,16,21,22,23,24]. The SOUND trial showed non-inferiority of omitting SLNB in patients with small tumours and negative AUS, with very low axillary recurrence rates [21]. The primary results of the INSEMA trial similarly support omission of axillary surgery in carefully selected cN0 patients with negative imaging, demonstrating non-inferior invasive disease-free survival and reduced surgical morbidity in the no-surgery arm [23].

However, most patients in these de-escalation trials did not receive NACT, and treatment decisions were not systematically stratified by molecular subtype or detailed receptor-defined risk. Furthermore, existing guidelines and expert consensus continue to recommend SLNB for cN0 patients after NACT, in part because residual nodal disease carries important prognostic and therapeutic implications, including indications for regional nodal irradiation and escalation of systemic therapy [16]. Our results extend the de-escalation discussion into the NACT setting and highlight the necessity of integrating tumour biology into imaging-based algorithms.

In patients with ultrasound-negative axillae after NACT—particularly those with luminal A and luminal B/HER2-negative disease—the false-negative rate and NPV of AUS observed in our study are too unfavourable to justify omission of SLNB at present. At the same time, it is important to interpret these diagnostic metrics in the context of modern systemic therapy and regional radiotherapy. Data from ACOSOG Z0011 and AMAROS suggest that not all residual nodal disease necessarily mandates extensive axillary surgery when appropriate adjuvant treatment is delivered [8,13]. Thus, the clinical impact of missing small-volume nodal disease on AUS remains uncertain and requires prospective evaluation, particularly in the NACT setting.

In contrast, patients with HER2-enriched or triple-negative tumours, who show high pCR rates under contemporary regimens, may in the future represent candidates for omission of SLNB if additional evidence confirms a very low residual nodal burden in the context of negative AUS and breast pCR [27,28,29,30,31]. Our data do not support such omission at this time, but they help to define the biological and imaging profile of subgroups in which prospective trials of post-NACT axillary de-escalation could be prioritised. The ROC-derived thresholds for ER, PR and Ki-67 identified in this study (82.5%, 25.0% and 17.5%, respectively) should be regarded as exploratory rather than definitive clinical cut-offs. They highlight an association between a strongly hormone-responsive, low-proliferative phenotype and a high probability of occult nodal disease in the ultrasound-negative axilla but require external validation. Future work should aim to integrate these biomarkers into multivariable risk models, potentially combined with radiologic or radiomic features from AUS and breast MRI, to provide more precise estimates of residual nodal risk.

### 4.4. Strengths and Limitations

This study has several strengths. First, it focuses on a clinically relevant yet under-represented population: breast cancer patients treated with NACT who have ultrasound-negative axillae. These patients are not well represented in existing de-escalation trials and are often managed according to evidence extrapolated from primary surgery cohorts. Second, we combined detailed clinicopathological data with quantitative imaging and immunohistochemistry and applied established statistical methods, including ROC-curve analysis and multivariable logistic regression, to identify independent predictors of SLNB positivity. Third, by analysing AUS performance both in the entire NACT-treated cohort and stratified by molecular subtype, we provide a more nuanced view of the heterogeneity of test performance across biological groups.

Nonetheless, several limitations should be acknowledged. The retrospective, single-centre design introduces potential selection and information bias, and the sample size—particularly within some molecular subtypes—is modest. This is reflected in the wide confidence intervals for several estimates and limits the precision of subtype-specific analyses and the complexity of multivariable models. As a result, the associations observed should be regarded as hypothesis-generating rather than definitive.

AUS examinations were performed and interpreted by different radiologists in a routine clinical setting, which may have contributed to variability in sensitivity and specificity; however, this heterogeneity likely reflects real-world practice and may enhance external relevance. Furthermore, SLNB and ALND pathology were not centrally reviewed, and we did not capture detailed information on systemic regimens, radiotherapy fields or breast pCR, all of which may influence nodal response and interact with tumour biology. Finally, long-term follow-up on axillary recurrence and survival was not available, precluding any conclusions regarding oncologic outcomes associated with different axillary management strategies after NACT.

### 4.5. Clinical Implications and Future Directions

Clinically, our findings support a biology-integrated approach to axillary decision-making after NACT. In patients with luminal A or luminal B/HER2-negative tumours and ultrasound-negative axillae, negative AUS alone should not be used to omit SLNB, given the high rate of occult sentinel node metastasis observed. In these subtypes, the presence of an intratumoural DCIS component and high PR expression further identifies patients at particularly increased risk of nodal involvement.

In contrast, for HER2-enriched and triple-negative subtypes with negative AUS after NACT, SLNB remains the standard of care but may, in the future, become a candidate for de-escalation in the context of response-adapted trials that incorporate breast and nodal pCR, comprehensive systemic therapy and optimised regional radiotherapy. Any such strategy will require robust prospective data demonstrating that omission of SLNB does not compromise axillary control or long-term survival.

Future research should therefore focus on: (i) validating our findings in larger, multi-centre cohorts with longer follow-up; (ii) integrating AUS findings, tumour biology and treatment response into multivariable risk models or nomograms to individualise axillary surgery; and (iii) prospectively evaluating the safety of omitting SLNB in selected biologically defined subgroups after NACT, with careful monitoring of axillary recurrence, distant failure and survival outcomes.

## 5. Conclusions

Preoperative axillary ultrasonography alone is insufficient to safely exclude sentinel lymph node metastasis in breast cancer patients receiving neoadjuvant chemotherapy, particularly in luminal A and luminal B/HER2-negative subtypes. In patients with ultrasound-negative axillae after NACT, the presence of an intratumoural DCIS component and high PR expression independently identify individuals at increased risk of SLNB positivity, while Ki-67 index provides additional, albeit less robust, information on residual nodal disease risk. These data indicate that ultrasound-negative axillae after NACT represent a biologically heterogeneous group in which tumour biology must be considered alongside imaging when planning axillary surgery.

Until prospective trials specifically address axillary de-escalation after NACT within biologically defined subgroups, SLNB should remain the standard staging procedure in luminal tumours despite negative AUS findings. In HER2-enriched and triple-negative subtypes, which exhibit higher nodal pCR rates, omission of SLNB may be explored only within carefully designed clinical trials that incorporate treatment response, systemic therapy and radiotherapy optimisation. Ultimately, integrating AUS, molecular subtype and therapeutic response into validated prediction tools may allow truly individualised axillary management while maintaining oncologic safety.

## Figures and Tables

**Table 1:** Baseline demographic and clinicopathological characteristics of the study cohort (*N* = 135).

Characteristic	Values
**Age, years**	48.49 ± 11.12
**Tumour laterality, *n* (%)**	
Left	67 (49.6)
Right	63 (46.7)
Bilateral	5 (3.7)
**Lesion pattern, *n* (%)**	
Solitary	114 (84.4)
Multiple	21 (15.6)
**Histopathological type, *n* (%)**	
Invasive ductal carcinoma (IDC)	55 (40.7)
IDC with ductal carcinoma in situ (DCIS)	62 (45.9)
Invasive lobular carcinoma (ILC)	3 (2.2)
ILC with DCIS	4 (3.0)
Others	11 (8.1)
**Presence of DCIS, *n* (%)**	
Absent	74 (54.8)
Present	61 (45.2)
**Microcalcification cluster on imaging, *n* (%)**	
Absent	59 (43.7)
Present	76 (56.3)
**Operation Type, *n* (%)**	
Breast-conserving surgery (BCS)	70 (51.9)
Mastectomy	65 (48.1)
**Axillary surgical approach, *n* (%)**	
Sentinel lymph node biopsy (SLNB)	75 (55.6)
Axillary lymph node dissection (ALND)	60 (44.4)
**Ultrasonographic tumour size, mm**	20.64 ± 13.48
**Pathologic tumour size, mm**	15.84 ± 9.85
**Nottingham histological score**	6.89 ± 1.1
**Lymphovascular invasion (LVI), *n* (%)**	
Absent	54 (40)
Present	81 (60)
**Oestrogen receptor (ER)**	59.61 ± 39.62
**Progesterone receptor (PR)**	28.84 ± 33.12
**Ki-67 proliferation index**	28.07 ± 25.53
**Her 2, *n* (%)**	
Negative	108 (80)
Positive	27 (20)
**Molecular subtype, *n* (%)**	
Luminal A	53 (39.3)
Luminal B/HER2−	34 (25.2)
Luminal B/HER2+	11 (8.1)
HER2-enriched	11 (8.1)
Triple-negative	26 (19.3)
**SLNB paraffin section, *n* (%)**	
Negative	66 (48.9)
Positive	69 (51.1)
**SLNB frozen section, *n* (%)**	
Negative	77 (57)
Positive	58 (43)
**Number of SLNs removed (frozen)**	3.87 ± 1.73
**Number of metastatic SLNs (paraffin)**	0.7 ± 1.03
**Number of SLNs removed (paraffin)**	4.77 ± 2.39
**Number of metastatic SLNs (frozen)**	0.90 ± 1.26
**Number of lymph nodes removed in ALND**	16.10 ± 7.08
**Number of metastatic lymph nodes in ALND**	3.60 ± 3.78
**Radiologic and pathologic concordance of the axilla, *n* (%)**	
Absent	96 (71.1)
Present	39 (28.9)

Radiologic–pathologic concordance was defined as agreement between preoperative axillary ultrasonography (reactive/non-suspicious vs. pathologic/suspicious) and final histopathologic nodal status (negative vs. positive). Data are presented as mean ± standard deviation (SD) for continuous variables and as number (percentage) for categorical variables. Abbreviations: IDC, invasive ductal carcinoma; DCIS, ductal carcinoma in situ; ILC, invasive lobular carcinoma; LVI, lymphovascular invasion; BCS, breast-conserving surgery; SLNB, sentinel lymph node biopsy; ALND, axillary lymph node dissection; ER, oestrogen receptor; PR, progesterone receptor; HER2, human epidermal growth factor receptor 2.

**Table 2:** Clinicopathological comparison between SLNB-negative and SLNB-positive patients in the axillary ultrasound-negative subgroup (*N* = 96).

Characteristic	SLNB− (*n* = 60)	SLNB+ (*n* = 36)	*p* Value
**Age, years, median (range)**	45 (27–70)	45 (32–72)	=0.124 ^U^
**Tumour laterality, *n* (%)**			=0.433 ^X2^
Left	25 (41.7)	19 (52.8)	
Right	33 (55)	15 (41.6)	
Bilateral	2 (3.3)	2 (5.6)	
**Lesion pattern, *n* (%)**			=0.190 ^X2^
Solitary	54 (90)	29 (80.6)	
Multiple	6 (10)	7 (19.4)	
**Histopathological Type, *n*(%)**			=0.145 ^X2^
IDC	32 (53.3)	12 (33.3)	
IDC + DCIS	20 (33.3)	21 (58.3)	
ILC	1 (1.7)	1 (2.8)	
ILC + DCIS	2 (3.3)	0 (0)	
Other	5 (8.3)	2 (5.6)	
**DCIS, *n* (%)**			**<0.001 ^X2^**
Absent	44 (73.3)	13 (36.1)	
Present	16 (26.7)	23 (63.9)	
**Microcalcification cluster, *n* (%)**			=0.062 ^X2^
Absent	30 (50)	11 (30.6)	
Present	30 (50)	25 (69.4)	
**Ultrasonographic tumour size, mm, median (range)**	15.5 (1–60)	12 (2–44)	=0.699 **^U^**
**Pathologic tumour size, mm, median (range)**	18 (2–80)	18 (4–55)	=0.958 **^U^**
**Nottingham histological score, median (range)**	7 (3–9)	6 (4–8)	**=0.005 ^U^**
**Lymphovascular invasion, *n* (%)**			=0.711 ^X2^
Absent	26 (43.3)	17 (47.2)	
Present	34 (56.7)	19 (52.8)	
**ER, %, median (range)**	70 (0–100)	90 (0–100)	**=0.003 ^U^**
**PR, %, median (range)**	1.5 (0–90)	40 (0–95)	**=0.003 ^U^**
**Ki-67, %, median (range)**	35 (1–90)	10 (2–90)	**=0.001** **^U^**
**HER2 status, *n* (%)**			**=0.005 ^X2^**
Negative	40 (66.7)	33 (91.7)	
Positive	20 (33.3)	3 (8.3)	
**Molecular subtype, *n* (%)**			**=0.003** ^X2^
Luminal A	17 (28.3)	21 (58.3)	
Luminal B/HER2−	10 (16.7)	10 (27.8)	
Luminal B/HER2+	9 (15)	1 (2.8)	
HER2-enriched	8 (13.3)	1 (2.8)	
Triple-negative	16 (26.7)	3 (8.3)	
**Number of SLNs removed, median (range)**	4 (2–13)	3 (1–7)	=0.218 ^U^

Data are presented as median (range) for continuous variables and as number (percentage) for categorical variables. Percentages for molecular subtypes are calculated within each SLNB status group (SLNB− and SLNB+). *p* values were calculated using the Mann–Whitney U test for continuous variables and Pearson’s chi-square test for categorical variables. In the table, the superscripts “^U^” and “^X2^” indicate the Mann–Whitney U test and Pearson’s chi-square test, respectively. Statistically significant *p* values (*p* < 0.05) are shown in bold. Abbreviations: IDC, invasive ductal carcinoma; DCIS, ductal carcinoma in situ; ILC, invasive lobular carcinoma; ER, oestrogen receptor; PR, progesterone receptor; HER2, human epidermal growth factor receptor 2; SLN, sentinel lymph node; SLNB, sentinel lymph node biopsy.

**Table 3:** (**A**). Diagnostic performance of preoperative axillary ultrasound for detection of axillary metastasis in the entire cohort (*N* = 135). (**B**). Diagnostic performance of axillary ultrasound for axillary metastasis according to molecular subtype.

(**A**)						
(a) Contingency table						
**Axillary Ultrasound Finding**		**Pathologic Axillary Metastasis** **Negative (*n* = 66)**		**Pathologic Axillary Metastasis** **Positive (*n* = 69)**		**Total (*n* = 135)**
Reactive/non-suspicious nodes		60 (44.4)		36 (26.7)		96 (71.1)
Pathologic/suspicious nodes		6 (4.4)		33 (24.4)		39 (28.9)
(b) Diagnostic performance indices						
**Parameter**				***n*** **/** ***N***	**Estimate, %**	
Sensitivity				33/69	47.8	
Specificity				60/66	90.9	
Positive predictive value (PPV)				33/39	84.6	
Negative predictive value (NPV)				60/96	62.5	
False-negative rate (FNR)				36/69	52.2	
Overall association (Pearson’s χ^2^, df = 1)				–	24.64 (*p* < 0.001)	
(**B**)						
**Molecular Subtype**	***n***	**Sensitivity, *n*/** ***N*** **(%)**	**Specificity, *n*/** ***N*** **(%)**	**PPV, *n*/** ***N*** **(%)**	**NPV, *n*/** ***N*** **(%)**	**FNR, *n*/** ***N*** **(%)**
Luminal A	53	14/35 (40.0)	17/18 (94.4)	14/15 (93.3)	17/38 (44.7)	21/35 (60.0)
Luminal B/HER2−	34	12/22 (54.5)	10/12 (83.3)	12/14 (85.7)	10/20 (50.0)	10/22 (45.5)
Luminal B/HER2+	11	1/2 (50.0)	9/9 (100.0)	1/1 (100.0)	9/10 (90.0)	1/2 (50.0)
HER2-enriched	11	2/3 (66.7)	8/8 (100.0)	2/2 (100.0)	8/9 (88.9)	1/3 (33.3)
Triple-negative	26	4/7 (57.1)	16/19 (84.2)	4/7 (57.1)	16/19 (84.2)	3/7 (42.9)

Values are given as *n* (% of the entire cohort). Axillary ultrasound categorised as “pathologic/suspicious” was considered a positive test result and “reactive/non-suspicious” as a negative test result. Diagnostic indices were calculated using final histopathologic nodal status as the reference standard (sensitivity = TP/[TP + FN], specificity = TN/[TN + FP], PPV = TP/[TP + FP], NPV = TN/[TN + FN], FNR = FN/[TP + FN]). Abbreviations: TP, true positive; FP, false positive; TN, true negative; FN, false negative; PPV, positive predictive value; NPV, negative predictive value; FNR, false-negative rate. Within each molecular subtype, axillary ultrasound categorised as “pathologic/suspicious” was considered a positive test result and “reactive/non-suspicious” as a negative test result. Sensitivity and specificity were calculated with final histopathologic nodal status as the reference standard (sensitivity = TP/[TP + FN]; specificity = TN/[TN + FP]). PPV and NPV were calculated as TP/[TP + FP] and TN/[TN + FN], respectively. FNR (false-negative rate) was defined as FN/[TP + FN] = 1 − sensitivity. Abbreviations: PPV, positive predictive value; NPV, negative predictive value; FNR, false-negative rate; HER2, human epidermal growth factor receptor 2.

**Table 4:** ROC analysis of ER, PR and Ki-67 for predicting SLNB positivity in patients with ultrasound-negative axilla (*N* = 96).

Biomarker	AUC (95% CI)	*p* Value	Optimal Cut-Off (% Positivity)	Sensitivity, %	Specificity, %
ER	0.679 (0.571–0.787)	0.003	82.5	58.3	63.3
PR	0.679 (0.571–0.788)	0.003	25.0	66.7	68.3
Ki-67	0.696 (0.587–0.805)	0.001	17.5	63.3	69.4

Data are derived from ROC-curve analyses performed in the 96 patients with preoperative axillary ultrasonography classified as reactive/non-suspicious (ultrasound-negative axillae). AUC, area under the ROC curve; CI, confidence interval; ER, oestrogen receptor; PR, progesterone receptor; SLNB, sentinel lymph node biopsy. Optimal cut-off values were selected using the Youden index.

**Table 5:** (**A**). Univariable logistic regression analysis for predictors of sentinel lymph node biopsy (SLNB) positivity in patients with negative preoperative axillary ultrasound (*n* = 96). (**B**). Multivariable logistic regression analysis of factors associated with sentinel lymph node biopsy (SLNB) positivity (*n* = 96).

(**A**)				
**Predictor**		**Comparison (İndicator vs. Reference)**	**OR (** **95%** **CI)**	***p*** **Value**
**Clinicopathologic variables**				
Age, years		Per 1-year increase	1.04 (0.99–1.08)	0.097
Pathologic tumour size, mm		Per 1 mm increase	1.01 (0.98–1.04)	0.743
DCIS component		Absent vs. present	0.21 (0.09–0.50)	<0.001
Microcalcification cluster		Absent vs. present	0.44 (0.18–1.05)	0.065
Lymphovascular invasion		Absent vs. present	1.17 (0.51–2.68)	0.711
Nottingham histological score		Low vs. high	3.27 (1.38–7.74)	0.007
**Biological markers**				
ER (%)		Low vs. high	0.41 (0.18–0.96)	0.041
PR (%)		Low vs. high	0.23 (0.10–0.56)	0.001
HER2 status		Negative vs. positive	5.50 (1.50–20.14)	0.010
Ki-67 index (%)		Low vs. high	3.93 (1.62–9.49)	0.002
**Molecular subtype** (reference: triple-negative)				
Luminal A		Luminal A vs. triple-negative	6.59 (1.64–26.43)	0.008
Luminal B/HER2−		Luminal B/HER2− vs. triple-negative	5.33 (1.18–24.21)	0.030
Luminal B/HER2+		Luminal B/HER2+ vs. triple-negative	0.59 (0.05–6.57)	0.670
HER2-enriched		HER2-enriched vs. triple-negative	0.67 (0.06–7.48)	0.742
(**B**)				
**Variable**	**Category (vs. Reference)**	**Adjusted OR**	**95% CI for OR**	***p*** **Value**
DCIS component	Absent vs. present	0.33	0.12–0.91	0.033
Microcalcification cluster	Absent vs. present	0.78	0.27–2.26	0.650
ER expression	Low vs. high	1.10	0.39–3.13	0.861
PR expression	Low vs. high	0.30	0.11–0.81	0.017
Ki-67 index	Low vs. high	2.60	0.96–7.05	0.060

Data are presented as odds ratios (ORs) with 95% confidence intervals (CIs) obtained from univariable binary logistic regression with SLNB positivity as the dependent variable (*n* = 96). For age and pathologic tumour size, ORs represent the change in odds of SLNB positivity per 1-year and per 1 mm increase, respectively. DCIS component, microcalcification cluster, lymphovascular invasion and Nottingham histological score were analysed as categorical variables, with the first category in the “Comparison” column coded as the indicator and the second as the reference. “Low” and “high” ER, PR and Ki-67 were defined according to ROC-derived cut-off values: ER low < 82.5% vs. high ≥ 82.5% positive tumour cells; PR low < 25.0% vs. high ≥ 25.0%; Ki-67 low < 17.5% vs. high ≥ 17.5%. HER2 status was dichotomised as negative (0/1+) versus positive (3+ or 2+ with gene amplification). Triple-negative tumours served as the reference category for all molecular subtype comparisons. OR > 1 indicates increased odds of SLNB positivity relative to the specified reference category. *p* values are based on Wald χ^2^ statistics. Abbreviations: SLNB, sentinel lymph node biopsy; DCIS, ductal carcinoma in situ; LVI, lymphovascular invasion; ER, oestrogen receptor; PR, progesterone receptor; HER2, human epidermal growth factor receptor 2; Ki-67, proliferation index; OR, odds ratio; CI, confidence interval. Values are adjusted odds ratios (ORs) with 95% confidence intervals (CIs) derived from a multivariable binary logistic regression model with SLNB positivity as the dependent variable (*n* = 96). All variables listed in the table were entered simultaneously into the model. The category shown first in the “Category (vs reference)” column was coded as the indicator level and the second as the reference. Thus, “absent vs. present” compares lesions without the specified feature to those with the feature, and “low vs. high” compares tumours with low biomarker expression to those with high expression. Cut-off values for “low” versus “high” expression were defined according to ROC-derived thresholds: ER < 82.5% vs. ≥82.5% positive tumour cells; PR < 25.0% vs. ≥25.0%; Ki-67 < 17.5% vs. ≥17.5%. OR > 1 indicates increased odds and OR < 1 decreased odds of SLNB positivity relative to the reference category. *p* values are based on Wald χ^2^ statistics. Abbreviations: SLNB, sentinel lymph node biopsy; DCIS, ductal carcinoma in situ; ER, oestrogen receptor; PR, progesterone receptor; Ki-67, proliferation index; OR, odds ratio; CI, confidence interval.

## Data Availability

The data presented in this study are available on request from the corresponding author. The data are not publicly available due to privacy and ethical restrictions.
